# Supplementary material for: Mutational analysis reveals a novel role for hepatitis C virus NS5A domain I in cyclophilin-dependent genome replication
Source: J Gen Virol. Author manuscript; Available in PMC 2024 Mar 5. (PMC7615712; doi:10.1099/jgv.0.001886)
Supplement: Supplementary material [file EMS194311-supplement-Supplementary_material.pdf]

(A)

JFH-1 mutant	Ratio WT/mutant Huh7 (48 hpe)	Ratio WT/mutant Huh7 (72 hpe)	Ratio WT/mutant Huh7.5 (48 hpe)	Ratio WT/mutant Huh7.5 (72 hpe)	CsA EC_{50} (nM)
WT	-	-	-	-	282.9
I52A	25.9	17.2	2.6	0.9	191.3
V67A	66	35.4	12.3	1.1	62.7
G70A	27.4	23.7	1.6	1.0	66.5
M72A	36.1	20.8	1.7	0.8	101.5
P141A	13.6	8.0	2.7	1.0	154.5
P145A	36.1	14.0	4.5	0.8	78.1
E148A	27.9	16.5	2.0	1.0	251.4

(B)

DBN3a mutant	Ratio WT/mutant Huh7 (48 hpe)	Ratio WT/mutant Huh7 (72 hpe)	Ratio WT/mutant Huh7.5 (48 hpe)	Ratio WT/mutant Huh7.5 (72 hpe)	CsA EC_{50} (nM)
WT	-	-	-	-	314.9
V52A	3.25	4.6	5.7	2.9	145.7
V67A	28.6	49	19	6.9	37.0
G70A	53	200	23	9.7	70.5
M72A	143	320	84	44	13.1
P141A	2	1.3	2.1	1.1	90.5
P145A	159	290	350	373	n/a
E148A	95	81	17.5	4.3	97.9

Supplementary Table 1: Comparison of SGR replication in Huh7 and Huh7.5 cells. Mean values for WT SGR replication taken from Fig. 2 (**A: JFH-1**) and Fig. 3 (**B: DBN3a**) at either 48 or 72 hours post electroporation (hpe) were divided by mean values for each mutant. Values close to 1 therefore indicate no impairment of replication for those mutants. Data show that for JFH-1 replication of the mutants is rescued in Huh7.5 cells compared to Huh7 at both timepoints **(A)**. However, the situation for DBN3a is not so clear cut – for most mutants the phenotype in Huh7 cells is maintained in Huh7.5 cells **(B).** CsA EC_{50} values measured in Huh7.5 cells from Figs 4A and 5A are shown for information.

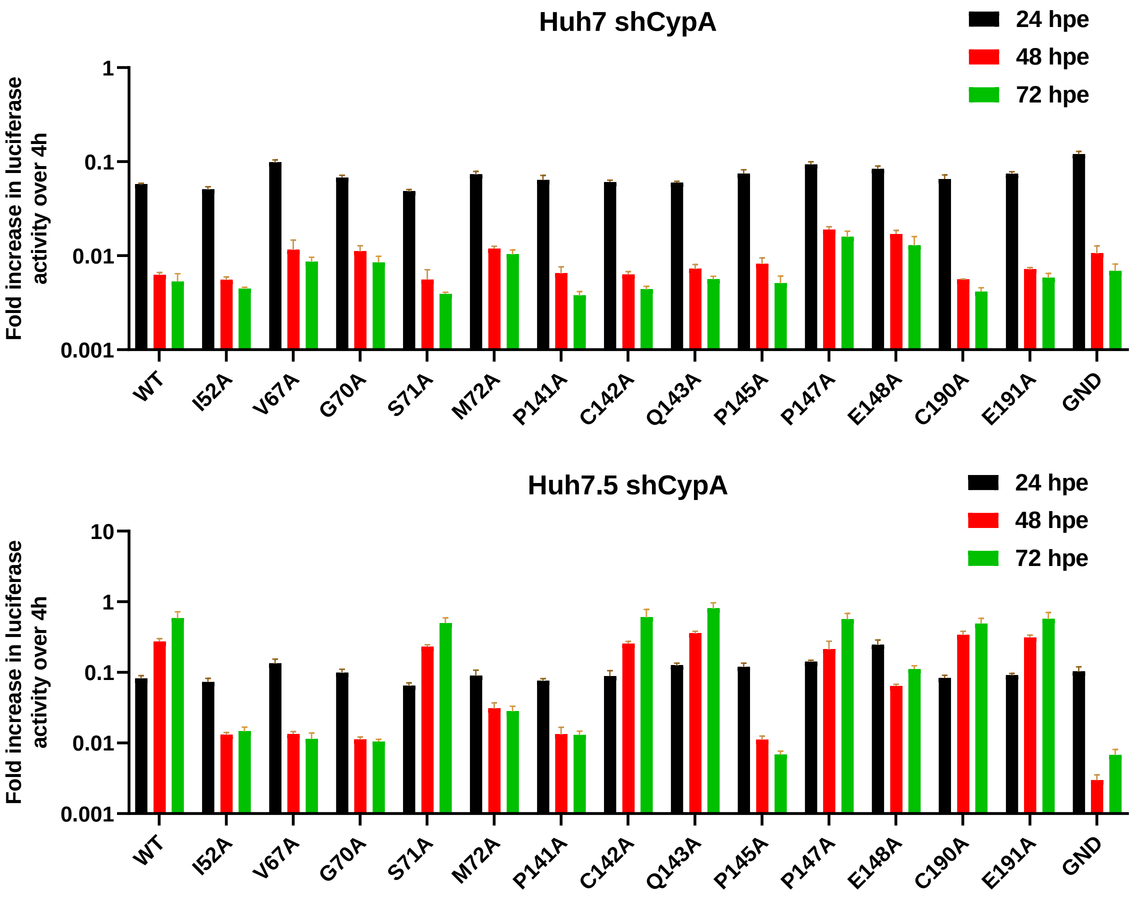

Supplementary Figure S1. SGR-luc-JFH-1 replication in CypA-silenced cells. The indicated cells were electroporated with SGR-luc-JFH-1 wildtype or mutant RNA and harvested at the indicated time points. Firefly luciferase activity was normalized with respect to 4 hpe.

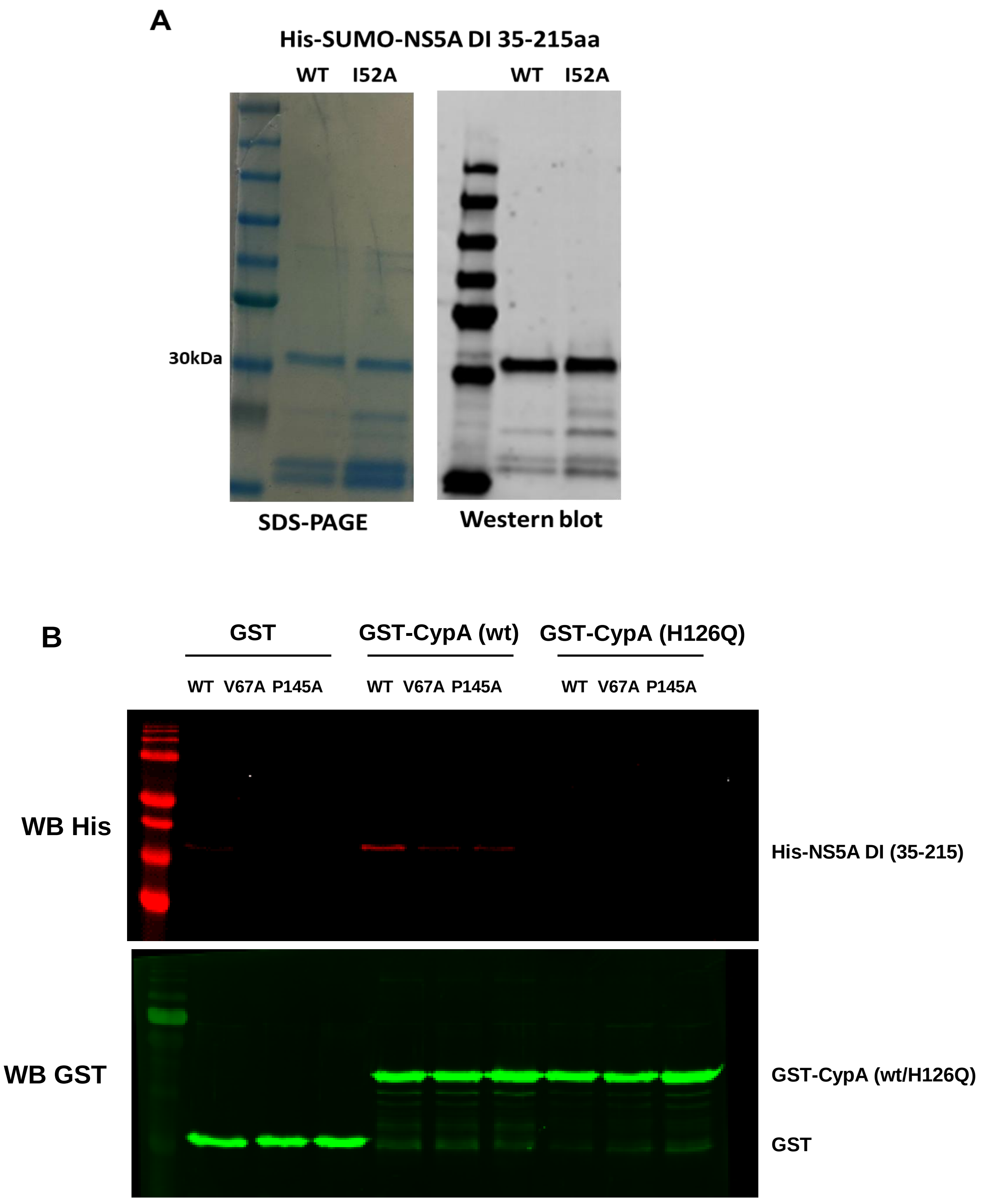

Supplementary Figure S2. Interaction between JFH-1 NS5A D1 and CypA. (A) Input prey protein – His-SUMO-NS5A D1 (amino acids 35-215) – wildtype and I52A as used in Fig 8c detected by Coomassie staining (left) or western blot with anti-His antibody (right). **(B)** GST, GST-CypA (wildtype) and GST-CypA (H126Q) were purified and bound to glutathione-agarose as a bait to precipitate purified His-SUMO tagged JFH-1 NS5A D1 (35-215) wildtype, V67A or P145A. The precipitates were analysed by western blotting for the His-tag (top), and inputs verified by GST western blotting (bottom).

```
      1                                                50
JFH1  SGSWLRDVWD WVCTILTDFK NWLTSKLFPK LPGLPFISCQ KGYKGVWAGT
DBN3a SGDWLRDIWD WVCTVLSDFK SWLSAKIMPA LPGLPFISCQ KGYKGVWRGD
Cons  SG.WLRD.WD WVCT.L.DFK .WL..K..P. LPGLPFISCQ KGYKGVW.G.

      51                                               100
JFH1  GIMTTRCPCG ANISGNVRLG SMRITGPKTC MNTWQGTFPI NCYTEGQCAP
DBN3a GVMSTRCPCG ATIAGHVKNG SMRLAGPRTC ANMWYGTFPI NEYTTGPSTP
Cons  G.M.TRCPCG A.I.G.V..G SMRL.GPKTC .N.W.GTFPI N.YT.G...P

      101                                              150
JFH1  KPPTNYKTAI WRVAASEYAE VTQHGSYSYV TGLTTDNLKI PCQLPSPEFF
DBN3a CPSPNYTRAL WRVAASSYVE VRRVGDFHYI TGATEDELKC PCQVPAAEFF
Cons  .P..NY..A. WRVAAS.Y.E V...G...Y. TG.T.D.LK. PCQ.P..EFF

      151                                              200
JFH1  SWVDGVQIHR FAPTPKPFFR DEVSFCVGLN SYAVGSQLPC EPEPDADVLR
DBN3a TEVDGVRLHR YAPPCKPLLR DDITFMVGLN SYAIGSQLPC EPEPDVSVLT
Cons  ..VDGV..HR .AP..KP..R D...F.VGLN SYA.GSQLPC EPEPD..VL.

      201                                              250
JFH1  SMLTDPPHIT AETAARRLAR GSPPSEASSS VSQLSAPSLR ATCTTHSNTY
DBN3a SMLRDPSHIT AETAARRLAR GSPPSEASSS ASQLSAPSLK ATCQTHRPHP
Cons  SML.DPSHIT AETAARRLAR GSPPSEASSS .SQLSAPSL. ATC.TH....

      251                                              300
JFH1  DVDMVDANLL MEGGVAQTEP ESRVPV---- LDFLEPMAEE ESDLEPSIPS
DBN3a DAELVDANLL WRQEMGSNIT RVESETKVVI LDSFEPLRAE IDDAELSVAA
Cons  D...VDANLL .......... ......---- LD..EP...E ..D.E.S...

      301                                              350
JFH1  ECMLPRSGFP RALPAWARPD YNPPLVESWR RPDYQPPTVA GCALPPPKKA
DBN3a ECFKKPPKYP PALPIWARPD YNPPLLDRWK APDYEPPTVH GCALPPRGAP
Cons  EC.......P .ALP.WARPD YNPPL...W. .PDY.PPTV. GCALPP....

      351                                              400
JFH1  PTPPPRRRRT VGLSESTISE ALQQLAIKTF GQPPSSGDAG SSTGAGAAES
DBN3a PVPPPRRKKT IQLDGSNVSA ALAALAEKSF PSSKPQEENS SSSGVDTQSS
Cons  P.PPPRR..T ..L..S..S. AL..LA.K.F .......... SS.G.....S

      401                                              450
 JFH1 GGPTSPGEPA PSETGSA.SS MPPLEGEPGD PDLESDQVEL QPPPQGGGVA
DBN3a ITSKVPPSPG GESDSESCSS MPPLEGEPGD PDLSCD---- ----------
Cons  .....P..P. ........SS MPPLEGEPGD PDL..D---- ----------

      451                    472
JFH1  PGSGSGSWST CS--EEDDTTV CC
DBN3a ------SWST VSDSEE-QSVV CC
Cons  ------SWST .S--EE....V CC
```

Supplementary Figure S3. Alignment of JFH-1 and DBN3a NS5A amino acid sequence. Cons: consensus identity. Dashes indicate where the sequences do not align and represent insertions. Residues bolded were subject to alanine scanning mutagenesis in this study.

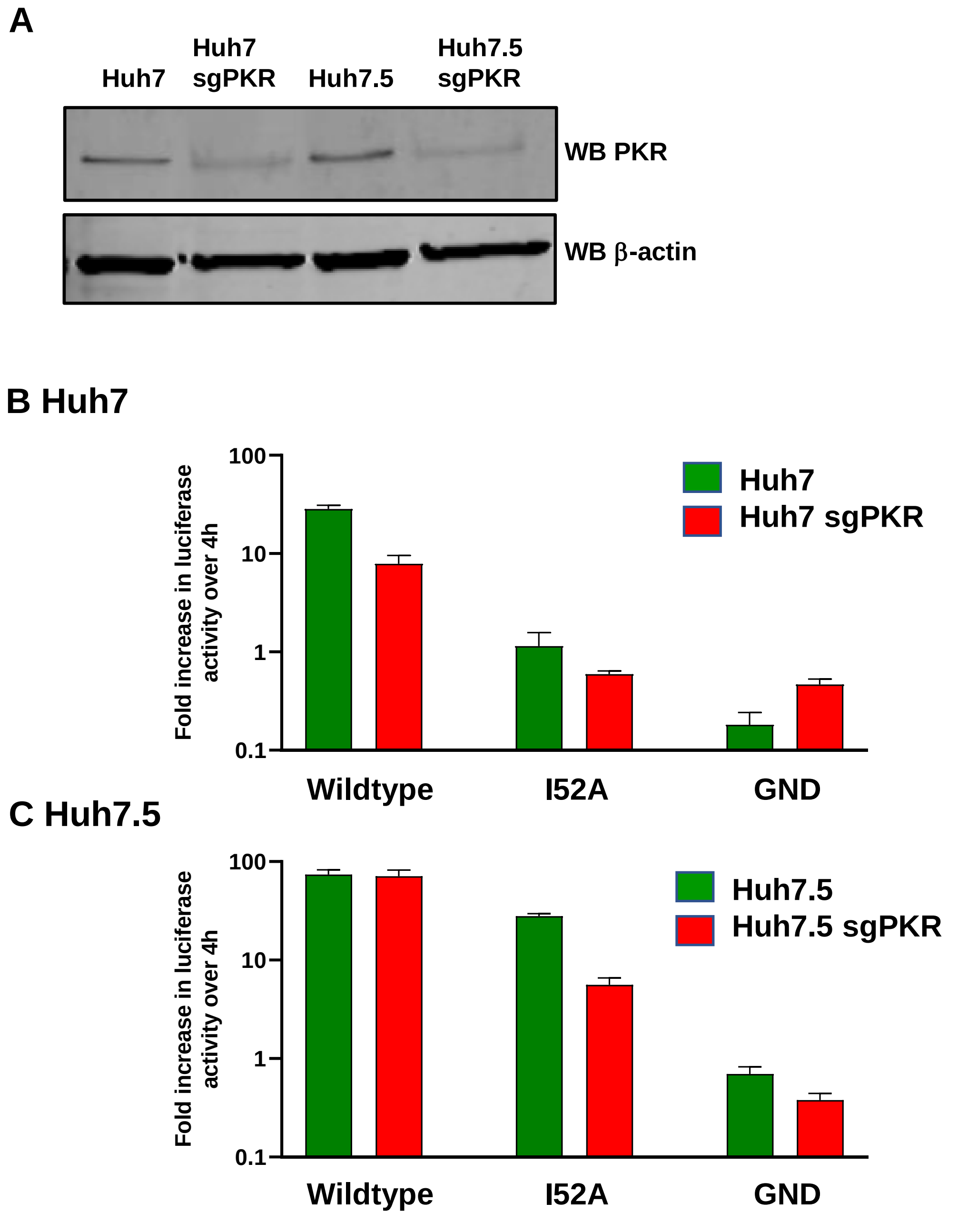

Supplementary Figure S4. SGR-luc-JFH-1 replication in PKR-silenced cells. A WB of control or PKR silenced cells. **B, C** The indicated cells were electroporated with SGR-luc-JFH-1 wildtype or I52A mutant RNA and harvested at 48 hpe. Firefly luciferase activity was normalized with respect to 4 hpe.

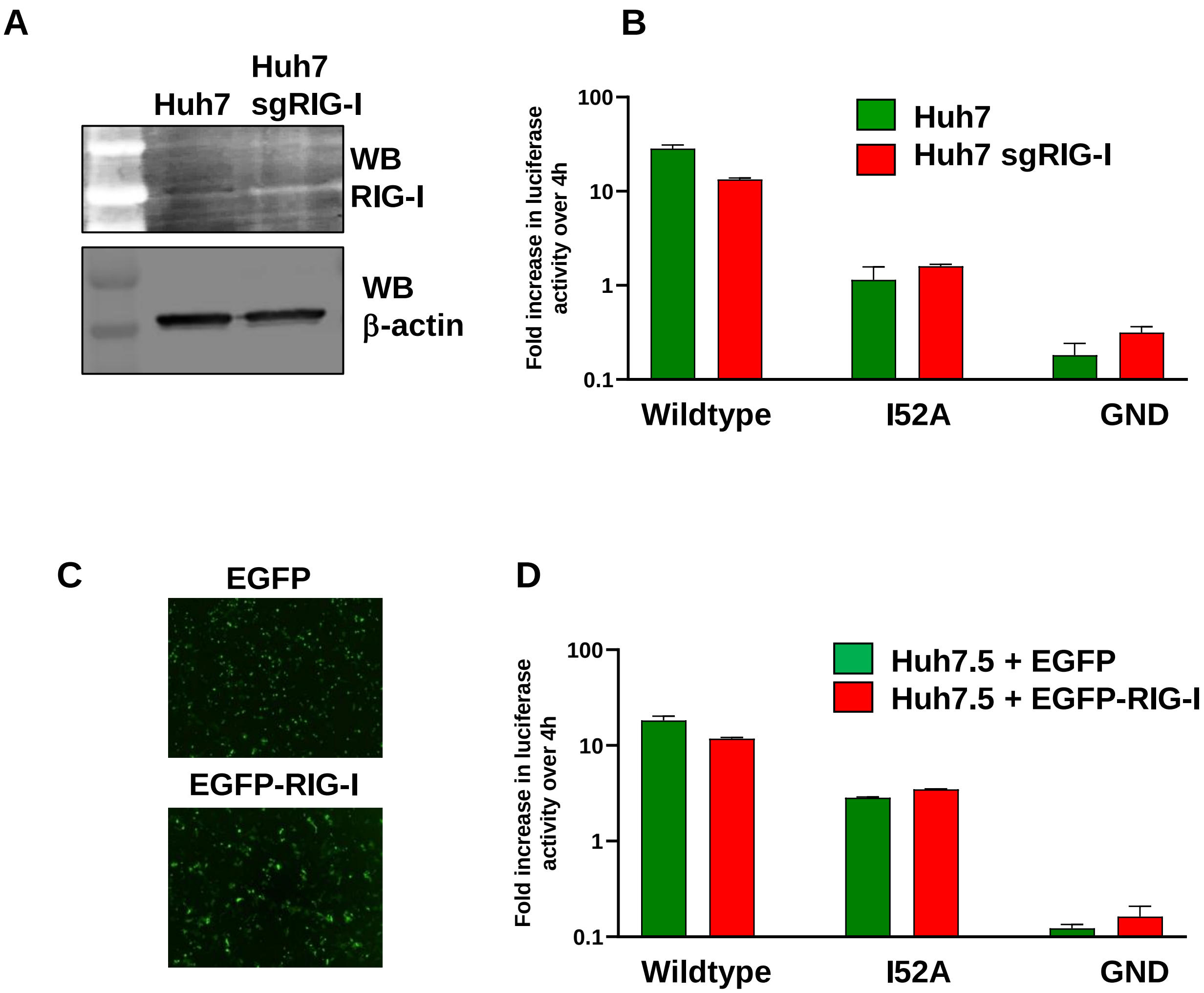

Supplementary Figure S5. SGR-luc-JFH-1 replication is not affected by RIG-I expression. **A** WB of control or RIG-I silenced Huh7 cells. **B** The indicated cells were electroporated with SGR-luc-JFH-1 wildtype or I52A mutant RNA and harvested at 48 hpe. Firefly luciferase activity was normalized with respect to 4 hpe. **C** Fluorescence microscopy of Huh7.5 cells transfected with pEGFP or pEGFP-RIG-I. **D** Huh7.5 cells transfected with pEGFP or pEGFP-RIG-I were electroporated with SGR-luc-JFH-1 wildtype or I52A mutant RNA and harvested at 48 hpe. Firefly luciferase activity was normalized with respect to 4 hpe.
